# Structure Reveals Regulatory Mechanisms of a MaoC-Like Hydratase from *Phytophthora capsici* Involved in Biosynthesis of Polyhydroxyalkanoates (PHAs)

**DOI:** 10.1371/journal.pone.0080024

**Published:** 2013-11-11

**Authors:** Huizheng Wang, Kai Zhang, Jie Zhu, Weiwei Song, Li Zhao, Xiuguo Zhang

**Affiliations:** 1 Department of Plant Pathology, Shandong Agricultural University, Tai’an, Shandong, China; 2 National Laboratory of Biomacromolecules, Institute of Biophysics, Chinese Academy of Sciences, Beijing, China; National Institute for Medical Research, Medical Research Council, London, United Kingdom

## Abstract

**Background:**

Polyhydroxyalkanoates (PHAs) have attracted increasing attention as “green plastic” due to their biodegradable, biocompatible, thermoplastic, and mechanical properties, and considerable research has been undertaken to develop low cost/high efficiency processes for the production of PHAs. MaoC-like hydratase (MaoC), which belongs to (*R*)-hydratase involved in linking the β-oxidation and the PHA biosynthetic pathways, has been identified recently. Understanding the regulatory mechanisms of (*R*)-hydratase catalysis is critical for efficient production of PHAs that promise synthesis an environment-friendly plastic.

**Methodology/Principal Findings:**

We have determined the crystal structure of a new MaoC recognized from *Phytophthora capsici*. The crystal structure of the enzyme was solved at 2.00 Å resolution. The structure shows that MaoC has a canonical (*R*)-hydratase fold with an N-domain and a C-domain. Supporting its dimerization observed in structure, MaoC forms a stable homodimer in solution. Mutations that disrupt the dimeric MaoC result in a complete loss of activity toward crotonyl-CoA, indicating that dimerization is required for the enzymatic activity of MaoC. Importantly, structure comparison reveals that a loop unique to MaoC interacts with an *α*-helix that harbors the catalytic residues of MaoC. Deletion of the loop enhances the enzymatic activity of MaoC, suggesting its inhibitory role in regulating the activity of MaoC.

**Conclusions/Significance:**

The data in our study reveal the regulatory mechanism of an (*R*)-hydratase, providing information on enzyme engineering to produce low cost PHAs.

## Introduction

MaoC-like hydratase (MaoC), possessing activity of (*R*)-specific enoyl-CoA hydratase ((*R*)-hydratase) involved in linking the β-oxidation and the polyhydroxyalkanoate (PHA) biosynthetic pathways, has been recently identified in the *fadB* mutant *Escherichia coli* strain [1]. MaoC is a new (*R*)-hydratase that catalyzes the (*R*)-specific hydration of the β-oxidation intermediate 2-trans-enoyl-CoA to (*R*)-3-hydroxyacyl-CoA [2,3]. Thus MaoC is an important enzyme in the biosynthesis of PHA because it supplies monomers that are subsequently polymerized to form PHA by PHA synthase [4,5]. PHAs are good candidates for “green plastic” due to their biodegradable, biocompatible, thermoplastic, and mechanical properties [6–9]. Because of this, considerable research has been undertaken to develop low cost/high efficiency processes for the production of PHAs [10–12].

To date a number of (*R*)-hydratases have been identified in both prokaryotes [4,13–15] and eukaryotes [16,17], all of which are known to accumulate PHA granules in cells. However, there are few reports of (*R*)-hydratases in oomycetes, so we select a worldwide soil borne disease *Phytophthora capsici* as a source organism for MaoC. The eukaryotic (*R*)-hydratases are similar in size, comprising about 300 amino acid residues with sequence identity ranging from 15% to 40% [18]. Additionally, the (*R*)-hydratases identified thus far show high catalytic efficiency for short-chain enoyl-CoAs (C_4_-C_6_) based on the fact that the resultant PHA is a copolymer of (*R*)-3-hydroxybutyric (C_4_) and (*R*)-3-hydroxyhexanoic (C_6_) acids [19]. Significant insight has been gained into the catalytic mechanism of (*R*)-hydratases by several solved crystal structures [20,21]. The structures show that (*R*)-hydratases have a conserved structural fold carrying the catalytic dyad, an aspartate and a histidine. The aspartate may function by activating a water molecule that attacks the substrate, whereas the histidine may act as a proton donor to substrate to promote the catalytic reaction. The multiple amino acid sequences alignment of hydratases has revealed the catalytic residues are located in a conserved region that is referred to as the hydratase 2 motif, recognizing as a motif Y-R-L-S-G-D-X-N-X-L-H-I-D-P-X-X-A. The hydratase 2 motif was first identified on human peroxisomal multifunctional enzyme type 2 [18], catalyzing a reaction by way of an acid-base mechanism. The motif is highly conserved at both sequence and structure levels [22]. Site-directed mutagenesis studies [16,17] indicated that another conserved residue, glycine (Gly-533 in 2-enoyl-CoA hydratase 2 from human peroxisomal multifunctional enzyme type 2, Hs-H2), is also important for the activity of (*R*)-hydratases. The substrate specificity was proposed to be conferred by the non-conserved portion located at the top of the substrate binding pocket [17].

As the environmental problem is becoming more and more serious all over the world, it is obviously significant to develop the “green plastic” to improve the environment. Here we report the crystal structure of a new enzyme involved in PHA biosynthesis.

## Results

### Protein purification and enzymatic activity assay of MaoC

**Figure 1:**
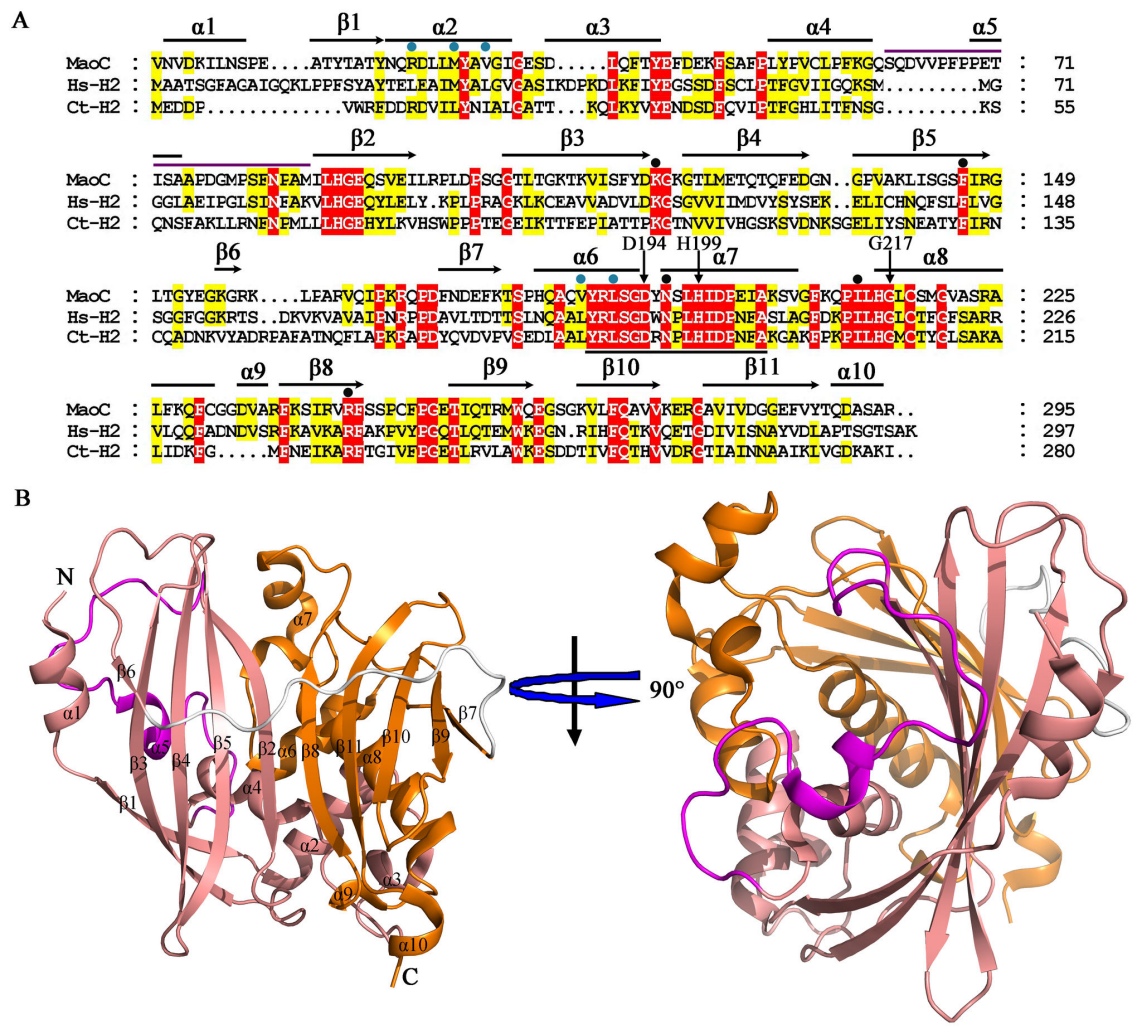
Overall structure of MaoC. (A) Sequence alignment of MaoC and its similar eukaryotic hydratases. Sequences used for the alignment are MaoC-like hydratase from *Phytophthora capsici* (MaoC), the corresponding parts of hydratase 2 regions of human (Hs-H2) and 2-enoyl-CoA hydratase 2 part of *Candida tropicalis* Mfe2p (Ct-H2). The corresponding SwissProt identifiers of MaoC, Hs-H2 and Ct-H2 are E3T2G3, P51659 and P22414, respectively. The secondary structure elements of MaoC, α-helices (black lines) and β-strands (black arrows), are marked above the sequence. Black vertical arrows indicate the catalytic residues of MaoC. The black line below the sequence alignment indicates the hydratase 2 motif, and the magenta line above the sequence alignment shows the regions of the hypothetical inhibitory segment of MaoC (residues 63-88). Residues participating in substrate binding and dimerization are labeled with black and cyan circles, respectively. (B) Different views of MaoC structure. The N- and C-domains, the intervening bridge, and the hypothetical inhibitory segment are colored in salmon, orange, white, and magenta, respectively. The two orientations of the structure are related by 90 ° rotations.

Sequence alignment (Figure 1A) showed that MaoC from *Phytophthora capsici* shares homology with hydratase 2 from human and *Candida tropicalis*. To study the enzymology of MaoC, we expressed His-tagged MaoC cloned into the vector of pET28 in *E. coli*. The expressed MaoC comprised about 40% of the total protein and was largely soluble. The yield of protein was about 60 mg per liter of culture. High expression of pET28-MaoC did not affect cell growth. Following affinity purification, MaoC was further cleaned by size exclusion chromatography. The finally purified MaoC was more than 95% pure based on an SDS–PAGE analysis showing a 36 kDa protein that is consistent with the predicted molecular weight of the protein. The activity of the purified MaoC was assayed by determining the hydration of crotonyl-CoA substrate as previously described [1]. The assay showed that the purified His-tagged MaoC exhibited enoyl-CoA hydratase activity, approximately 58.1 U/mg towards crotonyl-CoA.

### Overall structure of MaoC

To understand the molecular mechanisms underlying the regulation of MaoC, we set out to determine its crystal structure. For structural study, the full length MaoC was expressed in *E. coli* and purified as described above. MaoC was crystallized in the space group P2_1_2_1_2_1_ with two molecules (A and B) per asymmetric unit. The crystal structure was solved using molecular replacement with the coordinates of human 2-Enoyl-CoA Hydratase 2 (Protein Data Bank accession code, 1S9C) as the search model. The structure was refined to a resolution of 2.00 Å with *R*_work_ = 19.8% and *R*_free_ = 22.9%, respectively. Due to crystal packing, the two molecules differed slightly in their defined amino acids. In molecule A, no clear electron density was found for the two residues (Met-1 and Ser-2) from the N-terminal side and one (Leu-298) from the C-terminal side presumably because of their disordered structure in solution. In contrast, many additional residues (Glu-72 to Ala-77, Ser-83, Phe-84 and Ser-295 to Arg-297) from molecule B were not well defined.

The structure of MaoC is clearly divided into two domains, the N-terminal half (N-domain) and the C-terminal half (C-domain). Located between them is the intervening bridge region (Figure 1B). The N-domain (residues 3-159) is composed of five *α*-helices (*α*1 to *α*5) and six *β*-strands (*β*1 to *β*6), while the C-domain (residues 175-297) contains five *α*-helices (*α*6 to *α*10) and five *β*-strands (*β*7 to *β*11) (Figure 1B). In addition to the covalent connection by a loop (residues 160-174), the N- and the C-domains interact with each other through extensive non-covalent contacts. For example, the *β*2 from the N-domain and *β*8 from C-domain form a long anti-parallel *β*-sheet (Figure 1B).

As observed in the *β*-hydroxydecanoyl thiol ester dehydratase from *E. coli* [23], the *α*-helix *α*8 packs tightly against the six-stranded anti-parallel *β*-sheet, *β*7-*β*11, forming the core structure of the C-domain that is shaped like a hot dog (Figure S1). In addition, the C-domain contains a solvent-exposed loop structure (residues 192-215) with the amphipathic *α*-helices, *α*7 and *α*6 sandwiching the *α* *-*helix *α*8 and the *β*-sheet layer. The structure of the N-domain resembles that of the C-domain, although their sequences are not similar. However, they differ in several aspects. First, and perhaps the most striking, the core helix (*α*8) in the C-domain, which can be likened to the “sausage” of the hot dog fold, is replaced by a random coil connecting *α*4 and *α*5 in the N-domain; second, compared to the C-domain, the N-domain has an incomplete hot dog fold (Figure S1), comprising a four-stranded *β*-sheet, *β*2-*β*5 and lacking the long core *α*-helix; third, while the N-domain also contains a solvent-exposed structure (residues 32-52) comparable to the overhanging segment of the C-domain the *α*-helix (*α*3) in this region was not well defined.

### Structure comparison of MaoC with other hydratases

The DALI server [24] identified a number of structures similar to MaoC. The most closely related ones are 2-enoyl-CoA hydratase 2 from human peroxisomal multifunctional enzyme type 2 (Hs-H2) (PDB code 1s9c) [16] and the eukaryotic hydratase 2 in *C. tropicalis* (Ct-H2) (PDB code 1pn2) [17]. All three proteins form a conserved two-domain structure composed of an N-domain, a C-domain and an intervening bridge region between the two domains. Furthermore, each of the three structures has a hot dog fold core structure that is composed of a long and hydrophobic *α*-helix (“sausage”) packed against the anti-parallel *β*-sheet (“bun”). The extended *β*-sheet and the first part of the hot dog helix from the MaoC dimer are both well conserved in these three proteins. The similarity of the three structures resembles the homodimeric hot dog fold structures of prokaryotic (*R*)-hydratase and *β*-hydroxydecanoyl thiol ester dehydrase [17]. All three proteins form homodimers with a conserved structural organization (Figure S2). For structure comparison, the r.m.s.d. between MaoC and Ct-H2 is 1.903 Å for 477 C^*α*^ atoms, and the r.m.s.d. between MaoC and Hs-H2 is 1.688 Å for 487 C^*α*^ atoms.

**Figure 2:**
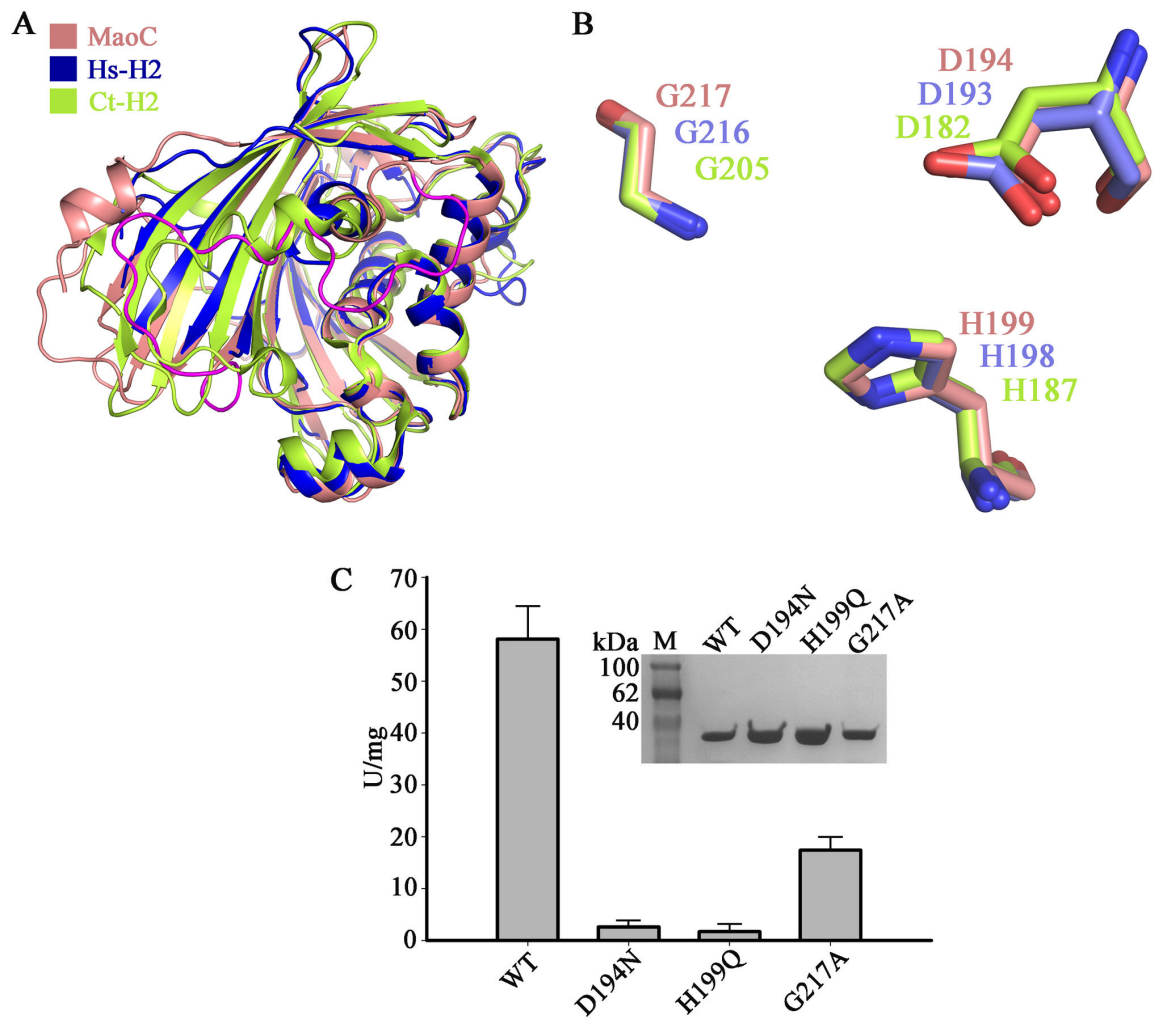
The active site of MaoC. (A) Superposition between MaoC and other similar structures. The structures used for the comparisons are colored as follows: MaoC, salmon; Hs-H2, blue; Ct-H2, lemon yellow. The major difference distinguishing MaoC from similar structures is the hypothetical inhibitory segment marked in magenta. (B) Superposition of the catalytic triad from MaoC (salmon), Hs-H2 (blue) and Ct-H2 (lemon yellow). The carbons, nitrogens, and oxygens of MaoC, Hs-H2 and Ct-H2 are colored in salmon, blue and red; slate, blue and red; lemon yellow, blue and red, respectively. (C) Mutations of the catalytic triad abolished the enzymatic activity of MaoC. The purified native MaoC and three mutants (upper), Enoyl-CoA hydratase activity for MaoC and its variants detected by crotonyl-CoA substrate (lower). The activities were assayed by determining the hydration of crotonyl-CoA substrate. One unit of activity was defined as the removal of 1 µmol of crotonyl-CoA per minute. The specific activity was defined as the activity of per milligram protein. The specific activities of the purified mutants were: D194N, 2.6 U/mg; H199Q, 1.7 U/mg; G217A, 17.4 U/mg, respectively. Mean of three independent experiments was considered.

One salient structural difference of MaoC from the other hydratases is that MaoC possesses an extra loop (residues 63-88) at the top of the substrate binding site. Sequence alignment (Figure 1A) revealed that this region is highly variable among hydratases. In addition, the structural segment immediately after *a*5 was not well superimposed among the three hydratases, further supporting a previous suggestion [17] that this region may be a structural determinant for substrate specificity. Other structural differences among the three structures reside in the loop regions such as residues Ser-63 to Met-88 (Figure 2A). The connections between *β*-strands in the hot dog fold family show a great variation [25]. The loop of MaoC joining *β*2 and *β*3 in the N-domain is significantly longer than other loops between the *β*-strands. The loop between *β*3 and *β*4 is much longer than those from Hs-H2 and Ct-H2.

### Active site and mutagenesis analysis

The attempts to crystallize MaoC in complex with its substrate failed. However, structure and sequence alignment indicated that the residues Asp-194, His-199 and Gly-217 of MaoC are equivalents of Asp-182, His-187 and Gly-205 in Ct-H2 and Asp-193, His-198 and Gly-216 in Hs-H2 (Figure 2B), respectively. In the latter two proteins, the three residues have been shown to be important for their catalytic activity. These data suggest that Asp-194, His-199 and Gly-217 of MaoC are catalytically crucial. To examine the importance of Asp-194, His-199 and Gly-217 for catalysis, three mutants, D194N, H199Q, and G217A, were made and purified to their homogeneity (Figure 2C). The purified mutant enzymes were confirmed by western blotting. As anticipated, these three mutations abolished the activity of MaoC with different levels (Figure 2C). CD spectra showed profiles similar to the wild type protein (Figure S3), indicating the reduced activities for these mutations are not due to structural perturbation.

### Functional significance of the dimeric MaoC

**Figure 3:**
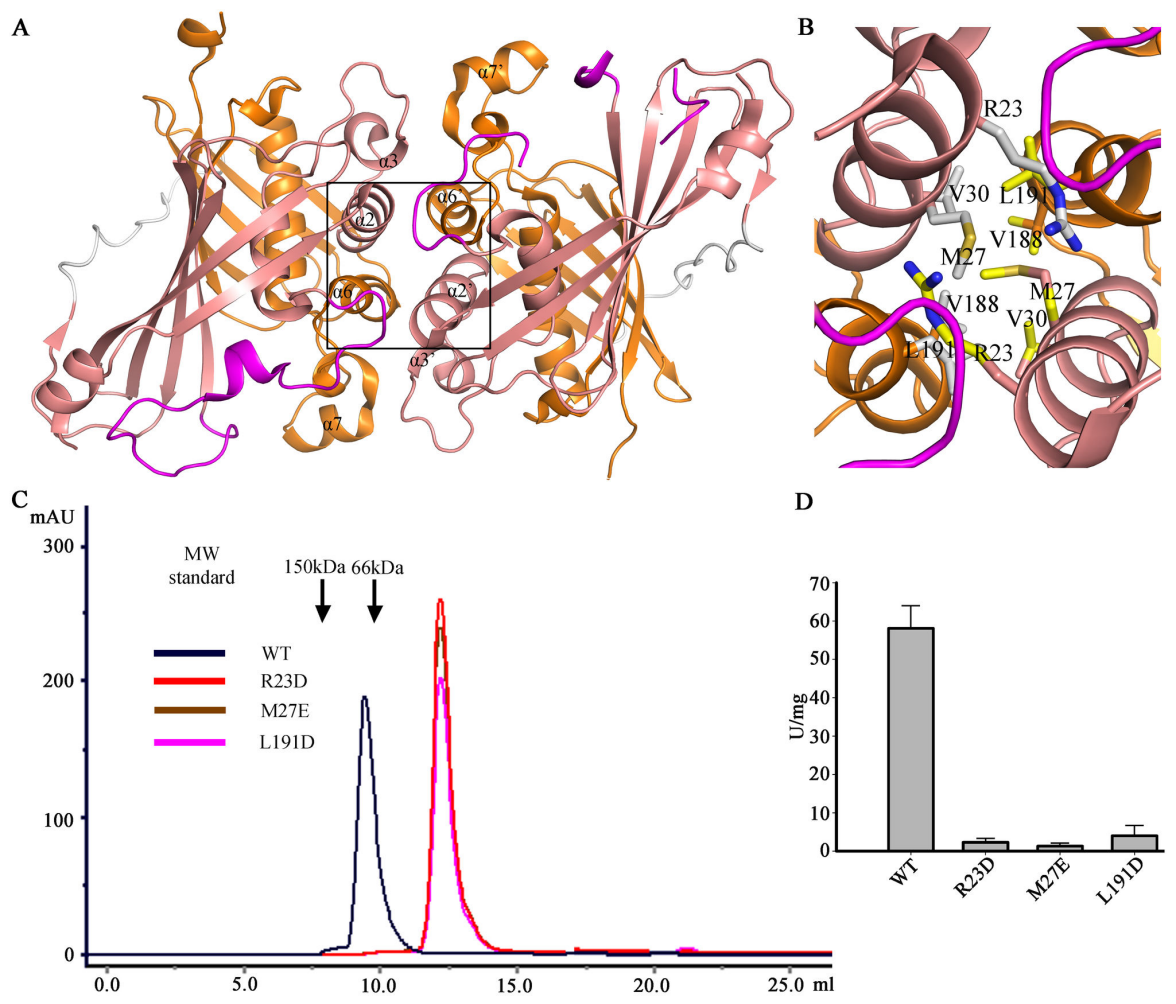
Dimerization is required for the enzyme activity of MaoC. (A) MaoC forms a homodimer in crystals. The key *α*-helices participating in dimerization (*α*2 and *α*2’, *α*6 and *α*6’, *α*3 and *α*3’, *α*7 and *α*7’) are labeled. The contacts of the central four-helix bundle are highlighted in a black frame. (B) The detailed dimeric interactions of the central four-helical bundle. The carbons, nitrogens, and oxygens of the side chains from the two monomeric MaoC are colored in gray, blue and red (Chain A); yellow, blue and red (Chain B), respectively. (C) MaoC forms dimer in solution. Shown in the figure are the gel filtration assay profiles of native and three indicated mutant MaoC proteins. The molecular weights of standard proteins are shown. (D) Monomeric MaoC has no enzymatic activity. The assay is similar to that shown in Figure 2C.

In their structure, the two monomeric MaoCs within an asymmetric unit make extensive contacts with each other, burying 1316.4 Å^2^ of the surface area of the monomers. Interactions of the two MaoC monomers are mainly mediated by the packing of four *α*-helices from one monomer against four *α*-helices arranged in an opposite order from the other monomer. The interactions result in a buried surface area much greater than is required for protein-protein interaction [26,27], suggesting the functional significance of the dimeric MaoC. The pairwise arranged helices, *α*2 (residues 21-32) and *α*3 (residues 36-41) from the N-domain, *α*6 (residues 184-193) and *α*7 (residues 196-208) from the C-domain of one MaoC monomer pack antiparallely against their counterparts from the other monomer, forming three four-helix bundle structures (Figure 3A). Hydrophobic interactions mediated by contacts of Met-27, Val-30, Val-188 and Leu-191 with their neighboring residues constitute the core of the dimeric interface. In addition to hydrogen bonding with Arg-23 from the other monomer, Arg-23 also makes hydrophobic contacts with Leu-191 and the Cα atom of Gln-64. At one side of the hydrophobic core, a large number of polar interactions, formed between the residues from the solvent-exposed loop of the N-domain and those from the overhanging segment of the C-domain, further strengthen the MaoC dimerization. For example, a pair of salt bridges is formed between Arg-190 and Glu-42 (not shown). The amino acids involved in formation of the dimeric interface are conserved among (R)-hydratases (Figure 1A), suggesting that dimerization is a common mechanism for this protein family. In addition to the major contacts via the three four-helix bundles, dimerization of MaoC is also contributed to by interaction between the solvent-exposed loop of the N-domain and the overhanging segment of the C-domain (Figure 3A). Specifically, Arg-23, Met-27, Val-30, Val-188 and Leu-191 form extensive hydrophobic contacts with their neighboring residues from the other subunit (Figure 3B).

Our structural analyses strongly suggest that MaoC is dimeric. To assess the functional significance of dimerization, we quantified the molecular weight of MaoC using a gel filtration assay. As anticipated, the MaoC protein was eluted at the position of 10.0 ml, comparable to that of the standard protein having a molecular weight of 66 kDa, indicating that MaoC formed a dimer in solution (Figure 3C). To provide additional evidence for the functional dimer, we made mutations of the residues at the dimer interface and assayed the purified mutants (Figure S4) for their oligomerization status and enoyl-CoA hydratase activities. Compared to the wild type MaoC protein, three of the mutants, R23D, M27E and L191D that were predicted to disrupt the dimeric interface, were eluted from a size exclusion column at a position corresponding to the monomeric MaoC (Figure 3C). Further supporting the functionally dimeric MaoC, all three mutant MaoC proteins exhibited little activity of hydrolyzing enoyl-CoA (Figure 3D). Together, these results demonstrate that dimerization is required for MaoC activity.

### An inhibitory segment of MaoC

**Figure 4:**
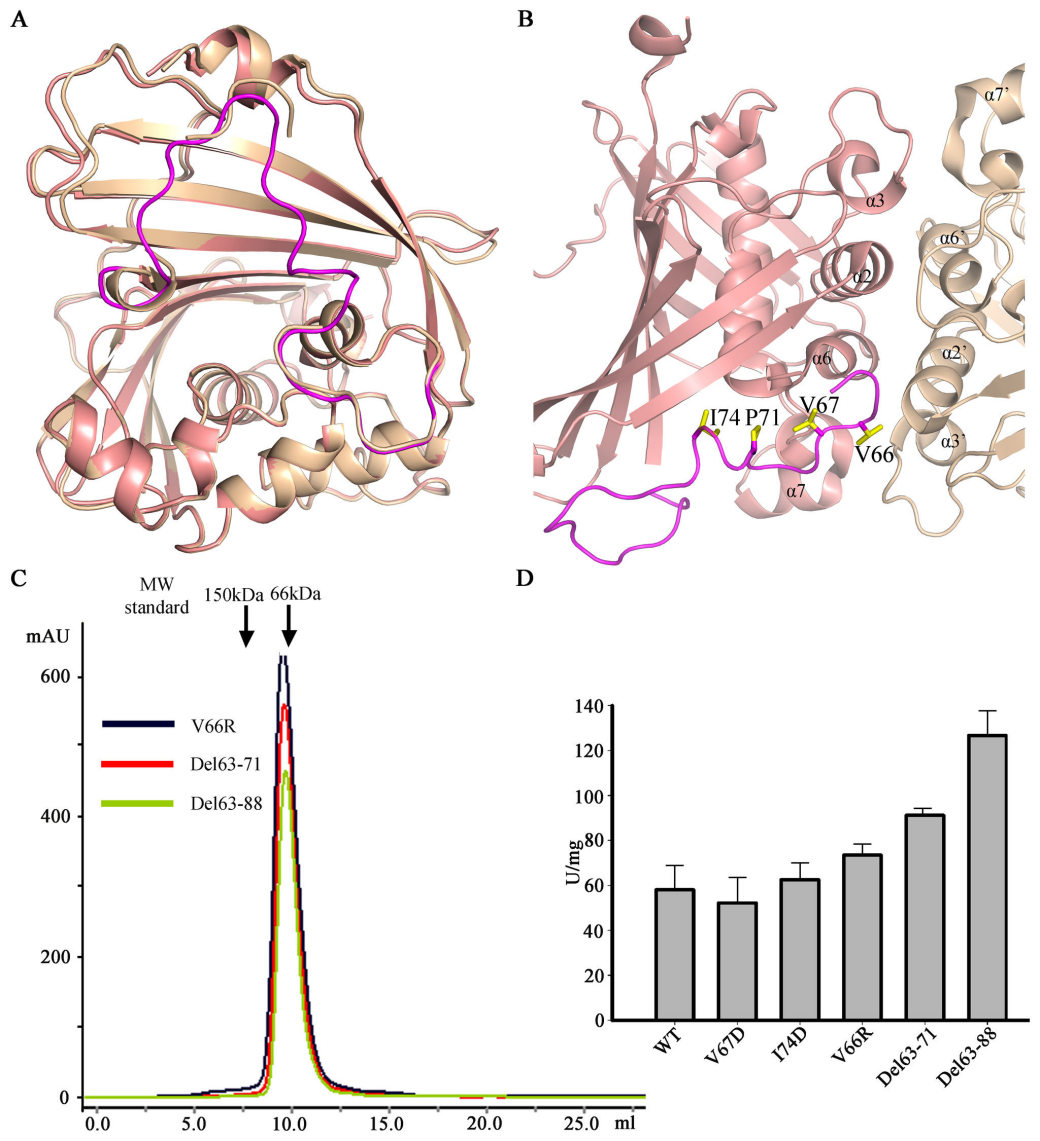
An inhibitory segment of MaoC. (A) Superposition of the molecule A (salmon) over B (wheat) of MaoC shows the inhibitor segment (magenta) in the molecule B is mostly broken. (B) The extra residues of MaoC (63-88aa) form a curved loop and make compact contacts with the *α*7. (C) Deletion of the non-conserved segment of MaoC has no effect on its dimerization. The assay is similar to that show in Figure 3C. (D) Deletion of the non-conserved segment of MaoC enhances its activity. The assay is similar to that shown in Figure 2C.

Structural comparison (Figure 4A) indicated that molecules A and B are nearly identical except that the residues 63-88 are well defined in A but disordered in B. Sequence alignment revealed that these residues constitute an extra segment in MaoC compared to other (*R*)-hydratases. In the dimer structure, the insertion was well defined in one MaoC monomer but not in the other. Structural examination indicated the well defined insertion is stabilized via crystal packing, suggesting that this region is flexible in solution. Interestingly, the stabilized insertion segment is located at the bottom of the tunnel of the active site in MaoC (Figure S5). The non-conserved hydrophobic amino acids mainly contribute to the interaction of the insertion region with the *α*-helix in MaoC that harbors the two catalytic residues (Figure 4B). This structural observation suggests that the non-conserved insertion may have a role in regulating the enzymatic activity of MaoC. If this is the case, deletion of the insertion would generate an effect on MaoC’s activity. To examine this possibility, we made single and deletion mutations surrounding the hypothetical inhibitory segment and then assayed their effect on MaoC’s activity. In full support of our prediction, one of the deletion mutant proteins, Del63-71, exhibited an activity of 91.2 U/mg in hydrolyzing enoyl-CoA, about 1.5 times that of the wild type protein. Further deletion of the amino acids around the insertion (Del63-88) produced a more striking effect on promoting MaoC’s activity (126.7 U/mg). The increased activity of the deletion mutant proteins was not caused by perturbation of the oligomerization status of MaoC, because they both were eluted at a similar position to the wild type protein in size-exclusion chromatography (Figure 4C). In contrast, single mutations in the insertion generated no significant effect on the activity of MaoC (Figure 4D). Collectively, our results indicate that an insertion region of MaoC plays a role in inhibiting its activity.

## Discussion

MaoC is a newly identified enoyl-CoA hydratase involved in the β-oxidation and polyhydroxyalkanoate (PHA) biosynthetic pathways [1]. Although the cloned *MaoC* shared 98% homology with the nucleotide sequence with that reported for it in JGI (The Joint Genome Institute, http://genome.jgi-psf.org/Phyca11/Phyca11.home.html), two amino acids differed between the deduced amino acid sequence and native *MaoC* from *P. capsici*. The difference may be attributed to strain diversity. In the present study, enzymatic activity assay using recombinantly purified MaoC confirmed that its enoyl-CoA hydratase activity is employed in the PHA biosynthetic pathway. The data from the assay support the conclusion that the enoyl-CoA hydratase is one of the most efficient enzymes when using the C4 substrate [20]. Because PHA-based plastics are potentially “green” substitutes for petroleum-based plastics, methodology that reduces the cost of producing PHA will be welcome. The high yield and efficiency of overexpressed MaoC in *E. coli* in the production of PHA offers such a methodology.

Similar to the eukaryotic hydratases, the C-domain of MaoC is in the form of a typical hot dog, with a large highly curved *β*-sheet wrapping around a long central *α*-helix. An incomplete hot dog fold of the N-domain may result from the lack of a long core *α*-helix. The hot dog fold was first observed for the *β*-hydroxydecanoyl thiol ester dehydratase from *E. coli* [23] and later for the 4-hydroxybenzoyl-CoA thioesterase from *Pseudomonas* sp. CBS-3 [28]. “Double hot dog” fold, meaning two hot dog repeats in a single peptide, has also been reported for the medium chain length acyl-CoA thioesterase II from *E. coli* [29]. To date the hot dog fold has been found in more than sixty proteins since the first report of its existence about a decade ago [22], hence the hot dog fold is an ancient and ubiquitous domain. The residues in the overhanging segment sandwiched between the strand *β*7 and the core helix *α*8 are highly conserved among the eukaryotic hydratase 2 proteins and MaoC, demonstrating their structural importance. Importantly, the catalytic triad, Asp-194, His-199 and Gly-217, are located on or near this segment, suggesting a functional significance for them in MaoC. In addition, residues from the substrate binding site recognizing the nucleobase ring are also conserved among different MaoC and other hydratases (Figure 1A). The highly conserved structural fold, catalytically important residues and substrate binding site suggest that MaoC and other hydratases may have been derived from a common ancestor protein.

In its structure, MaoC forms a homodimer that is mediated by four short *α*-helices from each monomeric MaoC. Interactions among the 8 helices result in formation of three 4-helical bundles, burying approximately 1316.4 Å^2^, a surface much larger than is required for protein-protein interaction [26,27]. These structural observations suggest that the dimeric MaoC is important for its function. Supporting this conclusion, mutations disrupting the dimerization of MaoC resulted in monomeric MaoC proteins in solution as indicated by a gel filtration assay. More importantly, the enzymatic activities were completely absent in the MaoC mutant proteins. Together, these data strongly support the notion that MaoC must be dimerized to function. The residues from the dimeric interface are invariable, suggesting that dimerization may be a conserved mechanism for their activity in the MaoC family of proteins. In support of this idea, most of the crystal structures of hydratases solved thus far are dimers with a similar interface to that in MaoC. But our study provides the first biochemical evidence for the functional significance of the dimer. A crystal structure of the monomeric MaoC will provide significant insight into the mechanism underlying the requirement of MaoC dimerization for its enzymatic activity. Unfortunately, all the efforts to crystallize a monomeric MaoC protein failed. Although future studies are needed to probe the mechanism, we propose that dimerization aligns the catalytic triad required for MaoC enzymatic activity (see further discussion below). A similar mechanism has been demonstrated in a Chlamydial protease [30].

Comparison of the structure of MaoC with the structure of other hydratases showed that they all have a highly conserved fold. Careful examination of structure revealed that the *α*7 harboring the catalytic residues Asp-194 and His-199 shifts outward about 0.5 Å as compared to other hydratases in either free or substrate-bound forms (not shown), while the catalytic residue Gly-217 can be well aligned among them. This may stem from the fact that the extra residues of MaoC form a curved loop and make compact contacts with the *α*7 (Figure 4B). The subtle structural difference in *α*7 of MaoC from other hydratases can result in a less well aligned catalytic triad of MaoC. Thus, our structural analyses suggest that the non-conserved residues of MaoC have an inhibitory role in the catalytic activity of MaoC. In complete agreement with this conclusion, deletion of the segment greatly promoted the enzymatic activity of MaoC. The non-conserved structure at the top of the substrate binding pocket has been suggested to be critical for substrate specificity. Our data show that a structural segment in MaoC has a role in negative regulation of its activity. It remains unclear how the MaoC activity is regulated in vivo. Nonetheless, our data raise the possibility that MaoC and probably other hydratases can be engineered to increase the production of PHA, thus lowering the costs for green plastics.

Despite many structural and biochemical studies on (*R*)-hydratases, little is known about how their enzymatic activity is regulated. In the present study, we solved the crystal structure of MaoC. The structure revealed that MaoC displays the canonical “hot dog” fold as observed in other (*R*)-hydratases and forms a homodimer. MaoC dimerization is mainly mediated by the conserved residues from the N- and C-terminal domain. In support of the functional significance of the dimeric MaoC, mutations of some of these residues led to disruption of MaoC dimer and a complete loss of its enzymatic activity in vitro, suggesting that dimerization is a conserved mechanism among (*R*)-hydratases that is required for catalysis. Compared to other (*R*)-hydratases, MaoC contains an extra loop capping an *α*-helix that integrates the catalytic dyad. Deletion of the loop resulted in noticeable enhancement of the enzymatic activity of MaoC, indicating that the loop has an inhibitory role in MaoC catalysis. Taken together, our data demonstrate the regulatory mechanisms for (*R*)-hydratases, providing valuable information about engineering MaoC for efficient PHA production.

## Materials and Methods

### Construction of the plasmid for overexpression

The full length of *MaoC* (GenBank Accession No. GU190361) was amplified by standard PCR from the genomic cDNA of *P. capsici* (SD33) using sense 5′-CGCGGATCCATGAGTGTGAACGTGGACAAG-3′ and antisense 5′-CCGGAATTCTTACAAACGCGCACTGGCGTC-3′ primers. The *Bam*HI and *Eco*RI restriction sites were designed into the sense primer and antisense primer, respectively. The purified PCR product was digested with *Bam*HI and *Eco*RI, and then subcloned into the expression vector pET28a (Novagen) to generate pET28-MaoC.

### Protein expression and purification

*E. coli* BL21 (DE3) pLysS cells carrying the recombinant plasmid were grown at 37°C in LB medium containing 50 ug/ml kanomycin until they reached an optical density at 600 nm of 0.5, and then induced with 1 mM IPTG for 20 h at 16°C.

Cells were harvested, resuspended in buffer A (10 mM Tris-HCl pH 8.5 containing 150 mM NaCl, 10 mM imidazole and 5 mM β-Mercaptoethanol) and lysed by sonication. The lysate was centrifuged at 15,000×g for 30 min at 4°C, the supernatant was loaded onto a Ni-nitrilotriacetic (Ni-NTA) Superflow column (GE Healthcare) equilibrated with buffer A linked to an ÄKTA purifier system (GE Healthcare). The column was washed with buffer B (buffer A containing 20 mM instead of 10 mM imidazole), buffer C (buffer B containing 0.5% (v/v) Tween-20) and buffer D (buffer A containing 40 mM instead of 10 mM imidazole), the proteins were eluted with buffer E (buffer A containing 200 mM instead of 10 mM imidazole). The solution was concentrated in a centrifugal concentrator (Millipore) and applied to a Superdex-75 gel-filtration column equilibrated in 10 mM Tris-HCl pH 8.5, 150 mM NaCl and 1 mM DTT. Fractions were collected according to their UV absorption peaks.

### Crystallization and data collection

Crystals of the enzyme were obtained by hanging drop vapour diffusion method in 100 mM HEPES pH 7.5, 6% (w/v) PEG6000, 5% (v/v) (±)-2-Methyl-2,4-pentanediol, as described previously [31]. The data were processed by HKL2000 [32]. Data processing statistics are shown in Table 1.

**Table 1:** Data collection and refinement statistics.

	**MaoC**
**Data collection**	
Space group	P2_1_2_1_2_1_
Cell dimensions	
*a*, *b*, *c* (Å)	81.458, 82.614, 124.228
*α*, *β*, *γ* (°)	90.0, 90.0, 90.0
Matthews coefficient (Å^3^ Da^-1^)	2.23
Solvent content (%)	45.0
Molecule in ASU	2
Resolution range (Å)	31.49-1.93 (1.96-1.93)^a^
Completeness (%)	97.9 (95.5)
*R*_merge_ (%)	11.1 (71.5)
Mean I/σ (I)	19.6 (2.6)
Redundancy	6.7 (5.9)
Wilson *B* factor (Å^2^)	38.4
**Refinement**	
Resolution range	31.49-2.00 (2.05-2.00)
No. of reflections	
all	57310
observed	53390
test set	2877
No. of atoms	5040
No. of residues	1124
No. of chains	3
*R*-factor	
All (%)	20.006
Observed (%)	20.006
*R*_work_/*R*_free_ (%)	19.8/22.9
No. of non-hydrogen atoms	
Protein atoms	4495
Solvent atoms	545
r.m.s.d.^b^ bonds (Å)	0.007
r.m.s.d. angles (°)	1.030
r.m.s.d. ∆*B* (bonded atoms) (Å^2^)	
All protein atoms	0.75
Main chain–Main chain	0.54
Side chain–Side chain	1.05
Main chain–Side chain	0.19
r.m.s.d. ∆*B* (Non-bonded contacts) (Å^2^)	
All protein atoms	2.14
Average *B* (Å^2^)	
All atoms	23.30
Main chain	23.03
Side chain	23.59
Solvent	35.96
Ramachandran plot (%)	
Most allowed	93.6
Additionally allowed	6.2
Generously allowed	0.0
Disallowed	0.2

a Values in parentheses are for highest resolution shell.

b r.m.s.d., room mean square deviations.

### Structure determination and refinement

The molecular replacement method was used to determine the structure of MaoC. An initial search model was generated from the coordinates of human 2-Enoyl-CoA Hydratase 2 (Protein Data Bank accession code, 1S9C) with the program Modeler7 [33]. Phaser [34] was used to find the unique top solution in the rotation and translation function and to calculate the optimal phases. Initial model building was carried out with ARP/wARP [35], and further model building was performed in COOT [36]. COOT was used to refine the model manually, combined with interactive restrained refinement by Refmac5.0 [37]. In the Ramachandran [38] plot 93.6% and 6.2% of the residues fell in favored and allowed regions respectively. The final model was refined to 2.00 Å resolution with *R*_work_ = 19.8% and *R*_free_ = 22.9%. Structure refinement statistics are summarized in Table 1. The atomic coordinates have been deposited in the Protein Data Bank (accession code 3KH8).

### Mutagenesis

Mutants were constructed using the MutanBEST Kit (Takara). The PCR primers (Table S1) containing mutational sites were designed in inverted tail-to-tail directions. Using the plasmids carrying the wild-type genes as templates, mutagenesis was performed according to the manufacturer’s instructions. The mutagenized plasmids were transformed into *E. coli* DH5α. Plasmids were isolated from the transformants and confirmed by DNA sequencing. *E. coli* BL21 (DE3) pLysS cells were then transformed with plasmids containing the verified mutations for protein expression. The mutational enzymes were expressed and purified to homogeneity essentially as described for the wild-type protein. The purified enzymes concentrations were confirmed spectrophotometrically by the Bradford method [39].

### Activity assay

The activities of the purified wild-type and mutated enzymes of MaoC were assayed by determining the hydration of crotonyl-CoA substrate. The purified wild-type and mutated enzymes were diluted to a final concentration of 1.0 mg/ml in 10 mM Tris–HCl (pH 8.5), 50 mM NaCl buffer. A 5 µl portion of an enzyme solution was added to 895 µl of 50 mM Tris-HCl (pH 8.0) containing 25 µM crotonyl-CoA, and the decrease in absorbance at 263 nm was measured at 30°C. The extinction coefficient (ε_263_) of the enoyl-thioester bond is 6.7×10^3^ M^-1^ cm^-1^ [40]. One unit of activity was defined as the removal of 1 µmol of crotonyl-CoA per minute. The specific activity was defined as the activity of per milligram protein. The assays were performed in triplicate.

### Circular dichroism (CD) spectrum analysis

The purified wild-type MaoC was diluted to a final concentration of 0.5 mg/ml in 10 mM Tris–HCl (pH 8.5), 50 mM NaCl buffer containing 1 mM DTT. CD spectra were measured by a Jasco J-715 spectropolarimeter and represent the mean molar ellipticity per amino acid residue of protein, and path length of cuvette used was 0.1 cm. Data were collected at 20 °C in a range of 190-240 nm in 0.5-nm intervals collecting data for 0.5 s at each point, and all spectra were baseline corrected. For each measurement ten spectra were used for accumulation. The structural conformations of the mutants were also evaluated by far-UV CD spectroscopy.

### Western blotting

To confirm the presence and the apparent molecular mass of the purified wild-type and mutant enzymes of MaoC, western blotting was carried out using anti-His antibody. Purified recombinant proteins were separated by 12% SDS–PAGE, electrotransferred onto a polyvinylidene fluoride (PVDF) membrane. The membrane was blocked with 5% (w/v) non-fat dry milk diluted in TBS buffer (10 mM Tris–HCl, pH 7.0, 100 mM NaCl) for 1 h, and then rinsed with TBS and placed in 1:5000 anti-His antibody for 2 h by constant shaking. The membrane was then washed with washing buffer (TBS buffer containing 0.5% (v/v) tween-20) three times (5 min each) and incubated with 1:1000 diluted horseradish peroxidase-conjugated anti-His monoclonal antibody (Tiangen) in TBS buffer for 1 h. Immune complexes were detected by enhanced chemiluminescence according to the manufacturer’s specifications.

### Analytical gel filtration chromatography

Analytical gel filtration chromatography of the purified wild-type MaoC or its various mutants (R23D, M27E, L191D, V66R, Del63-71, and Del63-88) were carried out on an ÄKTA purifier system (GE Healthcare). The purified enzymes were subjected to a 10/300 GL Superdex^TM^ 75 column preequilibrated with a buffer containing 10 mM Tris-HCl (pH 8.5), 100 mM NaCl and 1 mM DTT. Elution from the column was monitored by measuring absorbance at 280 nm. A calibration curve was generated by measuring the elution volumes of two standard proteins, albumin from bovine serum (BSA) and rabbit IgG, with a known molecular mass 66 kDa and 150 kDa, respectively. Molecular masses of the purified wild-type and mutational enzymes were estimated by interpolating their elution volumes onto the calibration curves.

## Supporting Information

Figure S1 **The monomer structure of MaoC contains two domains.** The C-domain forms a typical hot dog while the N-domain has an incomplete hot dog fold. The N and C-domains are colored in salmon and orange, respectively, and the exposed loops in white.
(TIF)

Figure S2 **Stereoview superposition of homodimers between MaoC and other similar structures.** The structures used for the comparisons are colored as follows: MaoC, salmon; Hs-H2, blue; Ct-H2, lemon yellow. The inhibitory segment that may further strengthen the MaoC dimerization is marked in magenta.
(TIF)

Figure S3 **CD spectra of the purified wild-type and mutants revealed no changes in secondary structure content.** CD spectra (D194N, H199Q, and G217A) showed profiles similar to the wild type protein, indicating the reduced activities for these mutations are not due to structural perturbation.
(TIF)

Figure S4 **Purification of the mutants.** The mutational enzymes were purified to homogeneity essentially as described for the wild-type protein.
(TIF)

Figure S5 **Overall view of the inhibitory segment.** The stabilized insertion segment is located at the bottom of the tunnel of the active site in MaoC. The carbons, nitrogens, and oxygens of the catalytic triad (D194, H199, and G217) are colored in salmon, blue and red, respectively; While the carbons, nitrogens, and oxygens of the other residues (V66, V67, P71, and I74) are colored in yellow, blue and red, respectively.
(TIF)

Table S1 **PCR primers used for construction of MaoC mutants.**
(DOC)
