# Supplementary figures and images for: Structure Reveals Regulatory Mechanisms of a MaoC-Like Hydratase from Phytophthora capsici Involved in Biosynthesis of Polyhydroxyalkanoates (PHAs)
Source: PLoS One. 2013 Nov 11;8(11):e80024. doi: 10.1371/journal.pone.0080024 (PMC3823801; doi:10.1371/journal.pone.0080024)

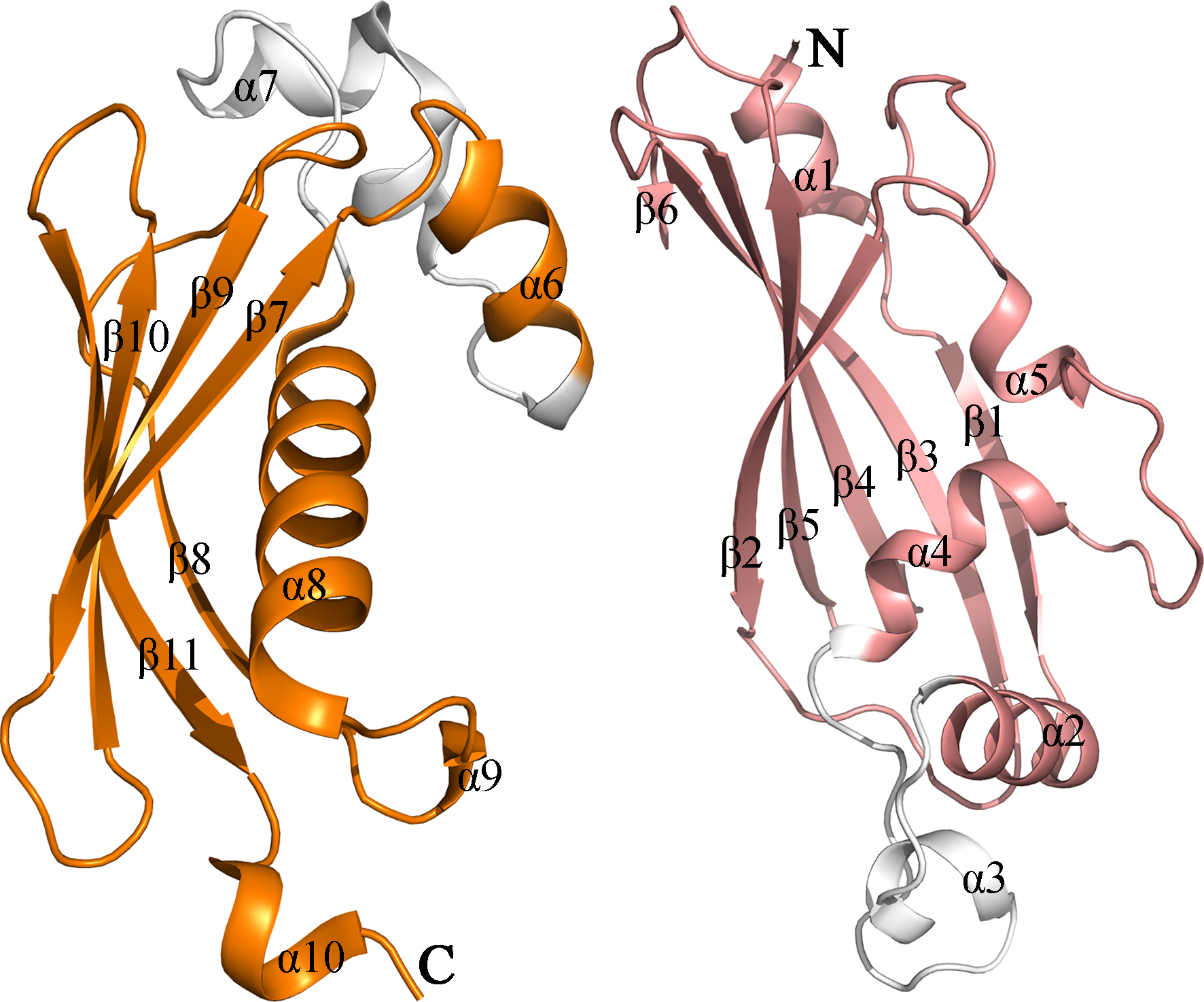

Supplement: Figure S1 — The monomer structure of MaoC contains two domains. The C-domain forms a typical hot dog while the N-domain has an incomplete hot dog fold. The N and C-domains are colored in salmon and orange, respectively, and the exposed loops in white. (TIF) [file pone.0080024.s001.tif]

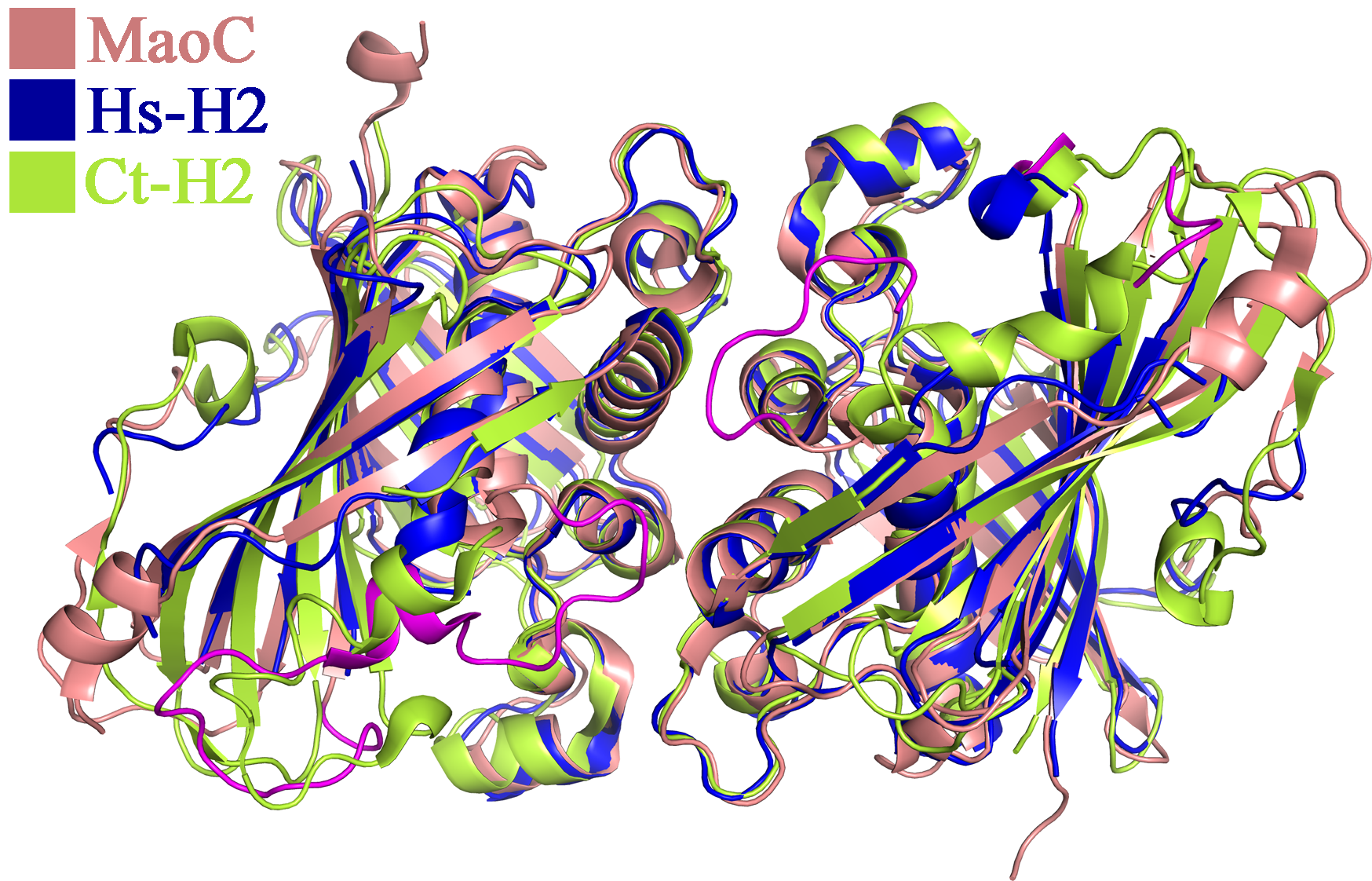

Supplement: Figure S2 — Stereoview superposition of homodimers between MaoC and other similar structures. The structures used for the comparisons are colored as follows: MaoC, salmon; Hs-H2, blue; Ct-H2, lemon yellow. The inhibitory segment that may further strengthen the MaoC dimerization is marked in magenta. (TIF) [file pone.0080024.s002.tif]

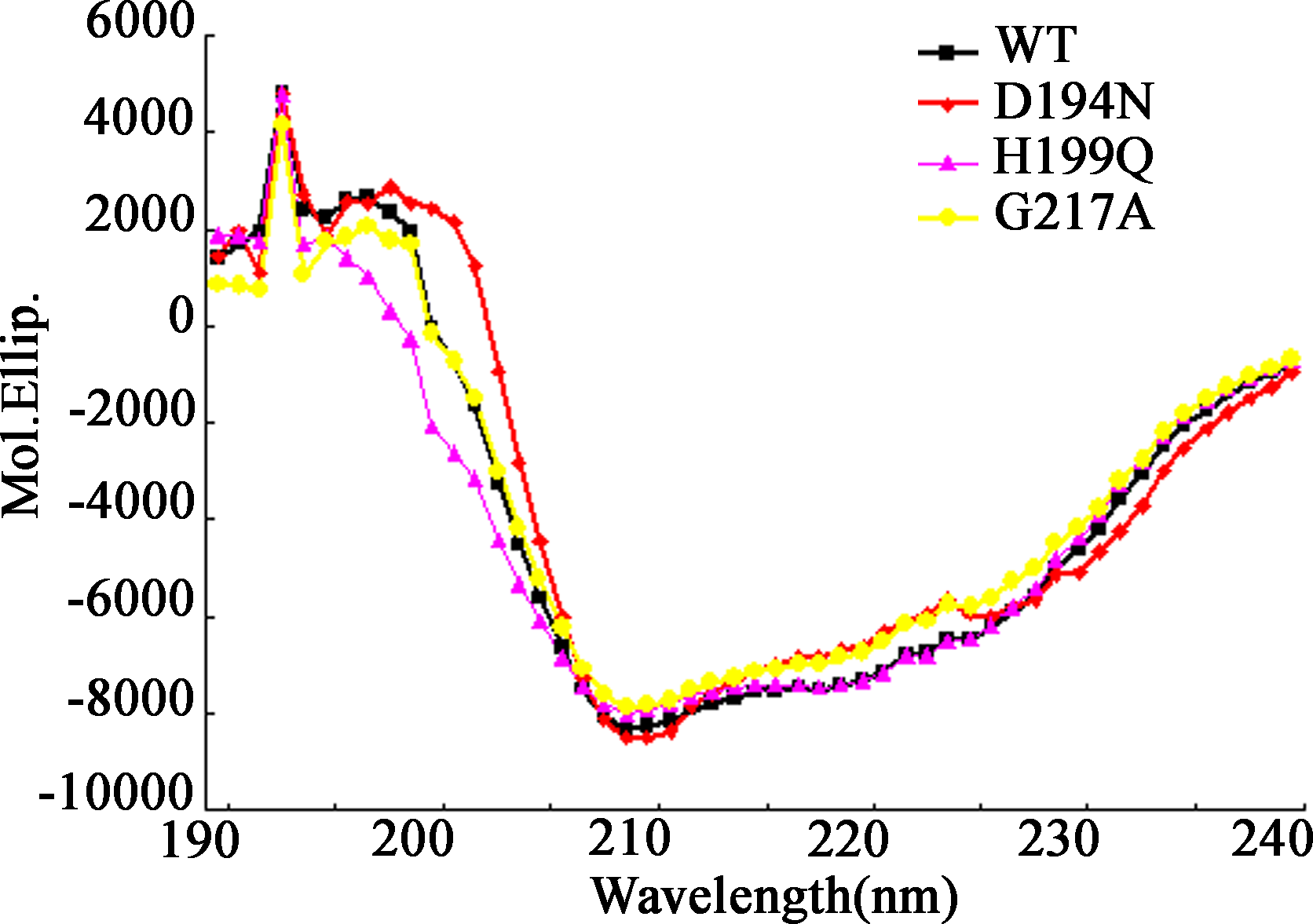

Supplement: Figure S3 — CD spectra of the purified wild-type and mutants revealed no changes in secondary structure content. CD spectra (D194N, H199Q, and G217A) showed profiles similar to the wild type protein, indicating the reduced activities for these mutations are not due to structural perturbation. (TIF) [file pone.0080024.s003.tif]

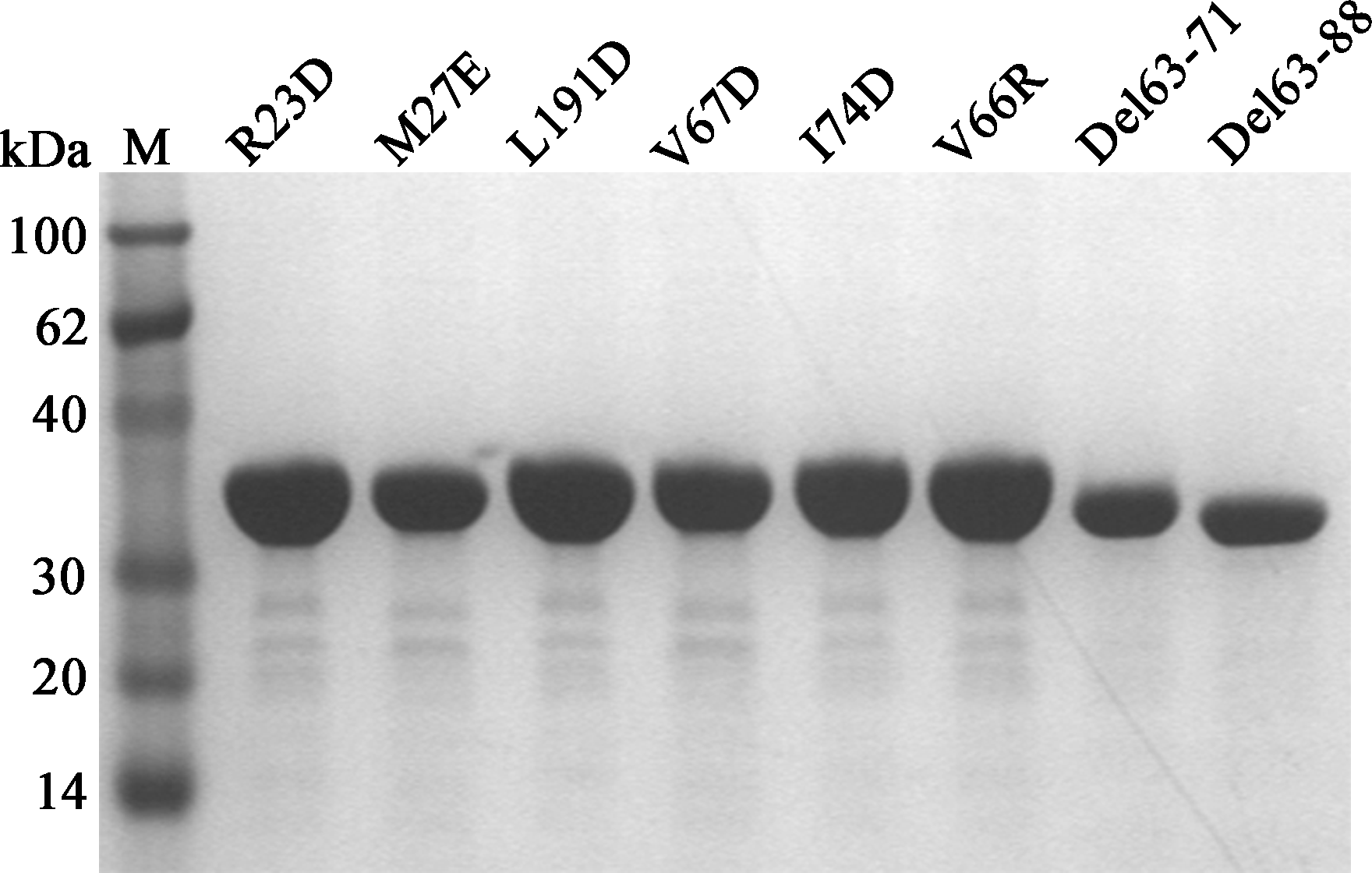

Supplement: Figure S4 — Purification of the mutants. The mutational enzymes were purified to homogeneity essentially as described for the wild-type protein. (TIF) [file pone.0080024.s004.tif]

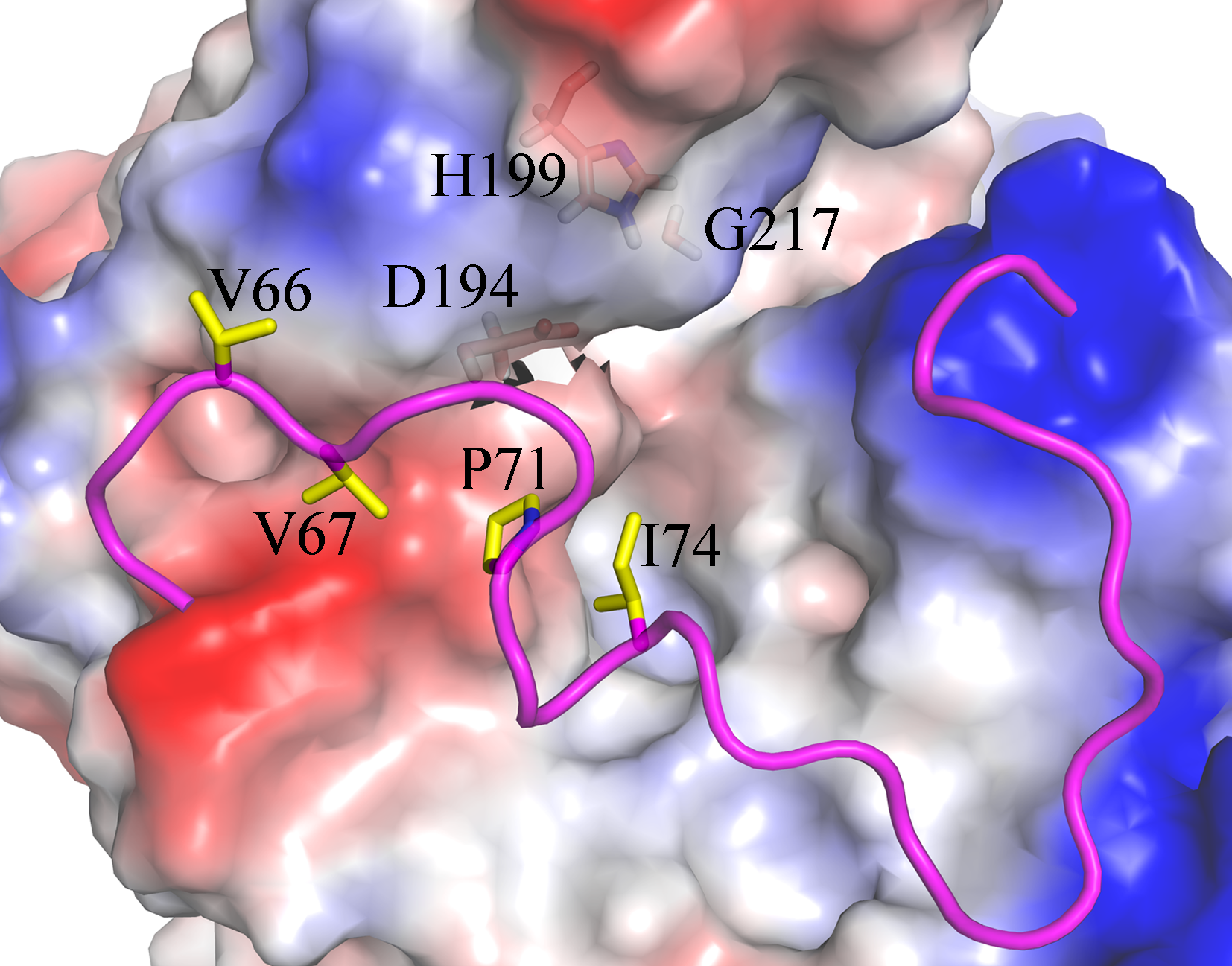

Supplement: Figure S5 — Overall view of the inhibitory segment. The stabilized insertion segment is located at the bottom of the tunnel of the active site in MaoC. The carbons, nitrogens, and oxygens of the catalytic triad (D194, H199, and G217) are colored in salmon, blue and red, respectively; While the carbons, nitrogens, and oxygens of the other residues (V66, V67, P71, and I74) are colored in yellow, blue and red, respectively. (TIF) [file pone.0080024.s005.tif]
