# Supplementary material for: Structure Reveals Regulatory Mechanisms of a MaoC-Like Hydratase from Phytophthora capsici Involved in Biosynthesis of Polyhydroxyalkanoates (PHAs)
Source: PLoS One. 2013 Nov 11;8(11):e80024. doi: 10.1371/journal.pone.0080024 (PMC3823801; doi:10.1371/journal.pone.0080024)
Supplement: Table S1 — PCR primers used for construction of MaoC mutants. (DOC) [file pone.0080024.s006.doc]

**Table S1.** PCR primers used for construction of MaoC mutants.

| Primer direction | Primer | Amino acid substitution | Sequence^a^ |
| --- | --- | --- | --- |
| Forward | MaoC |  | 5’-CGCGGATCCATGAGTGTGAACGTGGACAAG-3’ |
|  | D194N | Asp-194 to Asn | 5’-GTCGGGCAACTACAACTC-3’ |
|  | H199Q | His-199 to Gln | 5’-CTCGTTGCAGATTGATCCG-3’ |
|  | G217A | Gly-217 to Ala | 5’-CTATTCTGCACGCCCTGTG-3’ |
|  | R23D | Arg-23 to Asp | 5’-AACCAGGACGACCTGCTGA-3’ |
|  | M27E | Met-27 to Glu | 5’-CTGCTGGAATACGCGGTG-3’ |
|  | L191D | Leu-191 to Asp | 5’-GTCTACCGTGACTCGGGCGA-3’ |
|  | V67D | Val-67 to Asp | 5’-GATGTGGATCCATTTCCA-3’ |
|  | I74D | Ile-74 to Asp | 5’-CCACCTGAGACGGACTCAGCC-3 |
|  | V66R | Val-66 to Arg | 5’-AGGATCGGGTGCCATTTCCA-3’ |
|  | Del63-71 | Delete amino acids from 63 to 71 | 5’-GAGACGATCTCAGCCGCACCT-3 |
|  | Del63-88 | Delete amino acids from 63 to 88 | 5’-ATCCTGCACGGAGAGCAGTCG-3 |
| Reverse | MaoC |  | 5’-CCGGAATTCTTACAAACGCGCACTGGCGTC-3’ |
|  | D194N | Asp-194 to Asn | 5’-GAGTTGTAGTTGCCCGAC-3’ |
|  | H199Q | His-199 to Gln | 5’-CGGATCAATCTGCAACGAG-3’ |
|  | G217A | Gly-217 to Ala | 5’-CACAGGGCGTGCAGAATAG-3’ |
|  | R23D | Arg-23 to Asp | 5’-TCAGCAGGTCGTCCTGGTT-3’ |
|  | M27E | Met-27 to Glu | 5’-CACCGCGTATTCCAGCAG-3’ |
|  | L191D | Leu-191 to Asp | 5’-TCGCCCGAGTCACGGTAGAC-3’ |
|  | V67D | Val-67 to Asp | 5’-TGGAAATGGATCCACATC-3’ |
|  | I74D | Ile-74 to Asp | 5’-GGCTGAGTCCGTCTCAGGTGG-3’ |
|  | V66R | Val-66 to Arg | 5’-TGGAAATGGCACCCGATCCT-3’ |
|  | Del63-71 | Delete amino acids from 63 to 71 | 5’-CTGTCCTTTGAAGGGCAGGCA-3 |
|  | Del63-88 | Delete amino acids from 63 to 88 | 5’-CTGTCCTTTGAAGGGCAGGCA-3 |

^a^ The underlined sequences are the mutation sites.
